# Major differential gene regulation in *Coxiella burnetii* between in vivo and in vitro cultivation models

**DOI:** 10.1186/s12864-015-2143-7

**Published:** 2015-11-16

**Authors:** Runa Kuley, Ruth Bossers-deVries, Hilde E. Smith, Mari A. Smits, Hendrik I. J. Roest, Alex Bossers

**Affiliations:** Department of Infection Biology, Central Veterinary Institute part of Wageningen UR, Lelystad, The Netherlands; Department of Bacteriology and TSEs, Central Veterinary Institute part of Wageningen UR, Lelystad, The Netherlands; Host Microbe Interactomics, Wageningen University, Wageningen, The Netherlands

**Keywords:** *Coxiella burnetii*, Bacterial-transcriptomics, Virulence, Phase-variation, Mouse virulence bioassays, Gene regulation, Pathways, Metabolism, Adaptation

## Abstract

**Background:**

*Coxiella burnetii* is the causative agent of the zoonotic disease Q fever. As it is an intracellular pathogen, infection by *C. burnetii* requires adaptation to its eukaryotic host and intracellular environment. The recently developed cell-free medium also allows the bacteria to propagate without host cells, maintaining its infection potential. The adaptation to different hosts or extracellular environments has been assumed to involve genome-wide modulation of *C. burnetii* gene expression. However, little is currently known about these adaptation events which are critical for understanding the intracellular survival of *C. burnetii*.

**Results:**

We studied *C. burnetii* genome–wide transcriptional patterns in vivo (mice spleen) and in cell and cell-free in vitro culture models to examine its metabolic pathways and virulence associated gene expression patterns that are required to colonize and persist in different environments. Within each model, the gene expression profiles of the Dutch *C. burnetii* outbreak strain (602) and NM reference strains were largely similar. In contrast, modulation of gene-expression was strongly influenced by the cultivation method, indicating adaptation of the bacterium to available components. Genome–wide expression profiles of *C. burnetii* from in vitro cell culture were more similar to those seen for in vivo conditions, while gene expression profiles of cell-free culture were more distant to in vivo. Under in vivo conditions, significant alterations of genes involved in metabolism and virulence were identified. We observed that *C. burnetii* under in vivo conditions predominantly uses glucose as a carbon source (mostly for biosynthetic processes) and fatty acids for energy generation. *C. burnetii* experienced nutrient limitation and anaerobiosis as major stressors, while phosphate limitation was identified as an important signal for intracellular growth inside eukaryotic host cells. Finally, the in vivo environment significantly induced expression of several virulence genes, including those implicated in LPS synthesis, colonization, host component modulation and DNA repair mechanisms.

**Conclusion:**

Our study shows that *C. burnetii,* with its relative small genome, requires only a subset of core gene functions to survive under in vitro conditions, but requires the induction of full repertoire of genes for successful pathogenesis and thriving in harsh environments in vivo.

**Electronic supplementary material:**

The online version of this article (doi:10.1186/s12864-015-2143-7) contains supplementary material, which is available to authorized users.

## Background

Q fever, a worldwide zoonotic infectious disease, is caused by *Coxiella burnetii*, an intracellular Gram negative bacterium. Domestic ruminants (e.g., goat, sheep and cattle) are considered the main reservoir for Q fever infections in humans; the bacterium can cause a range of diseases depending on the host [1–3]. The main clinical manifestation of Q fever in goats and sheep are abortions, which result in the shedding of large numbers of bacteria into the environment. Inhalation of such contaminated aerosols is the main route of transmission in humans and can lead to acute or chronic Q fever [1, 4]. Acute infections range from asymptomatic to abrupt flu-like illness or pneumonia [5]; chronic infection is typically manifested as endocarditis [6]. An unprecedented outbreak of Q fever occurred in the Netherlands during the years 2007–2010, with more than 4000 human cases registered; infected dairy goats and sheep were identified as the primary source of the disease [7–11].

Attempts to understand the biology and pathogenesis of this bacterium through molecular techniques have been hampered by its intracellular lifestyle. However, a better understanding of the interactions of the pathogen with the host could be gained using global transcription profiles. This might aid in determining adaptations of the bacterium within the infected host and provide a fuller appreciation of its pathogenicity.

As an obligate intracellular pathogen, *C. burnetii* resides and proliferates within the acidic parasitophorous vacuole (PV) of the host cells [12]. The bacteria can infect a wide range of hosts and can also propagate to high quantities in different in vitro culture models [1, 13–15]. Within a laboratory setup, immune-competent mice are usually used as the in vivo animal model to assess the infection potential of *C. burnetii* [16]. The virulence of the strains is often measured based on splenomegaly and load of the bacteria in the spleen of mice [17–19]. *C. burnetii* are routinely cultured in BGM cells [15, 20], Vero cells [21] or macrophage like cell lines [12, 22]. The bacterium can establish persistent infections in cell culture, where it multiplies and survives in the cell PV. The latest advancement in Q fever research is the development of a host cell-free medium, designed based on the metabolic requirements of the bacterium. This complex nutrient medium supports substantial growth of most *C. burnetii* isolates and overcomes the drawbacks encountered when studying the pathogen in cell based culture or in vivo models [14, 15, 23].

Availability of complete genome sequences of *C. burnetii* contributed significantly towards understanding of the physiology and the pathogenic abilities of the bacterium [24]. However, information regarding environmental adaptations and expression of potential virulence factors important for bacterial colonization, replication and persistence under in vivo and in vitro systems is not entirely clear. Such information will provide insights into processes occurring during intracellular and extracellular growth of *C. burnetii* and allow direct evaluation of the relationship between gene expression patterns during growth inside living hosts and under in vitro conditions.

In this study, we endeavour to understand how the pathogen adapts to the host microenvironment, by monitoring the expression changes across the whole transcriptome of *C. burnetii* obtained within different propagation models. Main reservoirs of Q fever, such as pregnant goat and sheep, harbor the pathogen primarily in placentas, making this an ideal in vivo sample [20]. The bacterium has also been detected in oviducts and genital tissues of naturally infected non-pregnant goats [25]. However, use of these models to assess in vivo *C. burnetii* global transcriptional profiles is hampered by technical difficulties regarding the required fresh aborted sample collection (placentas), low RNA concentrations and quality. To avoid these limitations we used the spleens of experimentally infected mice as an in vivo model. We report here the global transcriptional pattern of *C. burnetii* from this in vivo model (mice spleen) compared with *C. burnetii* grown under laboratory conditions using cell-based and cell-free culture methods. In addition, we allowed strains to adapt from cell to cell-free culture propagation and considered the first two passages to determine the adaptation capabilities of the bacterium as it moves from one environment (propagation inside host cells) to another (cell-free cultivation). Our study provides a comprehensive view of *C. burnetii* metabolic adaptations and other important process required for its survival in response to diverse environments.

## Results and discussion

### Preparation of *C. burnetii* cells from different propagation models and measurement of its gene expression patterns

A whole-genome microarray was constructed based on all the available annotated genome sequences of *C. burnetii* in the databases. The mRNA levels of the expressed genes were assessed under in vivo and various in vitro conditions. For the in vivo transcriptome analysis, *C. burnetii* obtained from infected mice spleens were chosen, as spleens are highly responsive to infections and bacterial colonization in spleen is a good indicator of infectivity and persistence over time [17–19, 26]. During the time of sampling, all infected mice showed splenomegaly and an almost 10 fold increase in number of bacteria in the spleen. This spleen mice model offers several benefits, although it also has limitations, such as the usage of intraperitoneal (IP) infection route rather than intranasal route which is the best natural exposure model. However, because no significant changes in splenomegaly or bacterial numbers in spleens were observed between different inoculation routes, the IP route seems an easy and efficient inoculation route for *C. burnetii* infections in mice [27]. We determined the global transcriptional patterns of *C. burnetii* at 20 days p.i, because host immune responses towards bacterial infection were high around this period (data not shown) allowing us to examine bacterial metabolic adaptations, role of virulence factors and mechanisms to evade host defenses. *C. burnetii* infection in mice usually results in higher bacterial numbers in spleen at early infection stages (7–14 days) followed by significant decrease in bacteria around 20 days. This most likely indicates an influx of immune (T) cells into the spleen around 7 days p.i., resulting in increased splenomegaly and facilitating bacterial clearance thereafter [17]. To obtain sufficient bacterial RNA from spleens at day 20, a higher dose of bacteria (10^7^ genome equivalents) were inoculated in our mice. This high dosage was checked in our previous study, which showed that for mice given this dosage, splenomegaly and bacterial replication rate patterns at different time periods (Additional file 1: Figure S1) were similar to a lower dosage (10^4^ genome equivalents) [26]. Transcriptional profiles of *C. burnetii* from later stage of infection (20 days), where the bacterial numbers are significantly lower than at early infection stages, were compared with stationary phases of in vitro grown bacterium (cell and cell-free cultures) to examine environmentally-linked gene expression differences of the bacterium. To further study the various adaptations of the bacterium, *C. burnetii* propagated from first two serial passages in cell-free culture (p1 and p2) from cell-cultured strains and (fully) adapted cell-free cultured strains from stationary phase were considered. Gene expression of all cell-free cultured strains (p1, p2 and adapted) was compared with gene expression from cell culture as reference.

### Impact of cultivation conditions on *C. burnetii* transcriptome

**Fig. 1:**
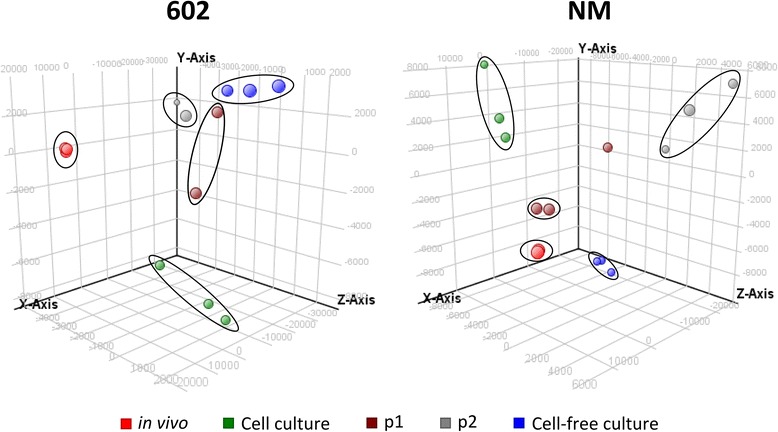
Principal component analysis on genome-wide expression profiles of *C. burnetii* strains 602 and NM obtained from various culture systems. Triplicate findings for each sample from different culture systems are clustered together indicative for experimental reproducibility. The plots of independent samples demonstrate also that based on their transcriptome profile, samples from different culture models can be differentiated

Good quality RNA was isolated from *C. burnetii* originating from different culture systems by depletion of the host RNA (except in cell-free culture system). The RNA was successfully labelled and used for microarray analysis (in triplicate). Principal component analysis (PCA) plots were constructed, which clearly showed that most sample triplicate clustered tightly, indicative of good experimental reproducibility of samples for each strain in different culture systems (Fig. 1).

### Niche specific gene expression patterns of *C. burnetii*

**Fig. 2:**
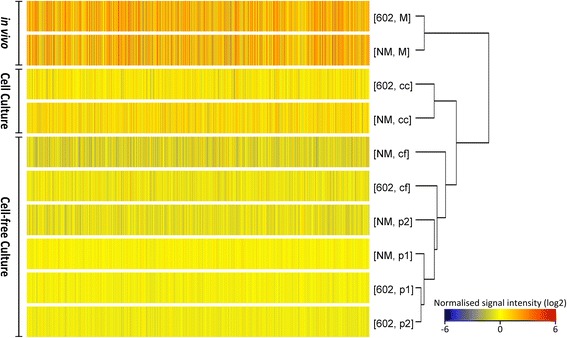
Hierarchical clustering analysis on normalised signal intensities of probes of 602 and NM strains in different culture models. Data shows clustering of in vivo and in vitro models separately based on patterns of gene expression. Normalised signal intensity (log2) of probes (average of triplicates) for each condition are represented as a colour scale from red for high expressions to blue for lower expressions. In vivo (M = mice spleen), cell culture (cc), cell-free adapting phase passage 1 (p1) and passage 2 (p2), cell-free culture model (cf)

To get an overview of the gene expression of 602 and NM strains induced by different culture models, hierarchical clustering was performed based on average normalised signal intensity of probes. Clustering showed that the expression profiles of strains are largely determined by the method in which they were cultured or isolated (Fig. 2). The expression profiles of *C. burnetii* isolated from in vivo and in vitro models clustered separately. Within the in vitro culture models, expression profiles of strains from cell culture were closer to the in vivo model, followed by expression profiles of strains obtained from the cell-free culture model. These results suggest that gene expression of *C. burnetii* depends on the available components present in the growth medium, tissue or cell. Hence, the possible differences in the metabolic capabilities of the strains are clearly seen in culture models considered in the study.

### The global transcription profile in different culture models

Because the different strains have transcriptional profile clustering based on the culture model in which it was propagated, thorough studies of gene expression were conducted. We determined which genes showed major differential expression in the spleens of mice compared to in vitro models. Moreover, we looked into differences in gene expression that existed between in vitro culture models. These detailed studies were conducted using the data from the Dutch outbreak strain 602, which was shown to be highly virulent by our previous mouse virulence bioassays [15]. Genespring microarray software analysis showed 906 of the 2061 genes were, compared to in vitro cell culture, differentially regulated (*p* < 0.05, fold change of 2) in 602 strains from mice spleen, with a majority of genes (67 %) up-regulated. Comparing expression profiles of the 602 strain under in vitro conditions (cell-free vs. cell culture model) showed that 438 genes were differentially regulated, but the majority of genes (71 %) were down-regulated. A complete microarray dataset from the different comparisons are presented as supplementary material (Additional file 2: Table S1).

**Fig. 3:**
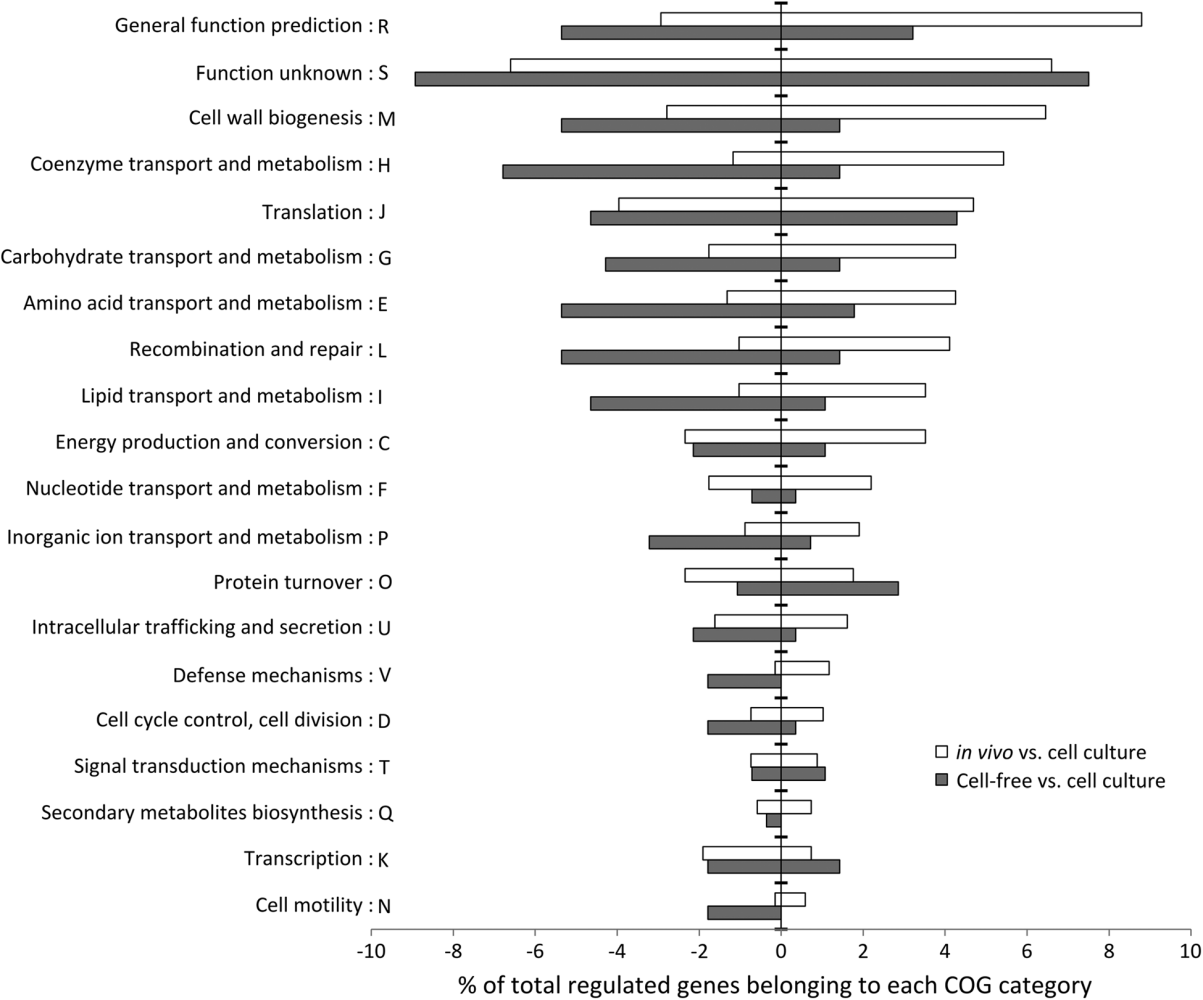
Functional COG-categories of differentially regulated *C. burnetii* genes under in vivo and in vitro culture models. Functional categories of regulated genes of the 602 strain under in vivo and cell-free culture compared with cell culture model. The up-regulated and down-regulated genes are shown on the right and left side of the y-axis respectively. Largest group of regulated genes in all culture models belonged to the unknown function category (S). Large number of up-regulated genes under in vivo conditions belonged to the metabolism group such as coenzyme, carbohydrate, amino acid and lipid transport and metabolism (HGEI). Whereas, the largest number of up-regulated genes in cell-free culture compared to cell culture belonged to the protein synthesis group (O)

Next, in various growth models, we determined the functional categories [28] and performed pathway enrichment analysis of the differentially regulated genes (Fig. 3 and Table 1). In the in vivo model, when compared with genes expressed in cell culture, with the exception of the group of unknown, hypothetical and conserved hypothetical proteins (44 % of in vivo induced genes), the largest group of functional category genes (HEGCIFPQ) and pathways showing increased expression belonged to the metabolism group (Fig. 3, Table 1). In contrast, fewer genes and pathways involved in metabolism were up-regulated in the cell-free culture model than in the cell culture model. Apart from the metabolic genes, other genes seen to be highly up-regulated in vivo when compared to cell culture belonged to defence mechanisms (V), cell wall biogenesis (M) and recombination and repair (L) categories probably due to bacterial resistance to the harsh environments of PV. In cell-free culture 16 % of induced genes belonged to translation (J) and post translational modifications/protein-turnover (O) categories indicating more protein synthesis leading to faster growth rates compared to other conditions.

**Table 1:** Significantly regulated (KEGG) Pathways in different culture models of *C. burnetii*

Pathway ID	Pathway	m vs. cc	p1 vs cc	p2 vs. cc	cf vs cc
	Metabolic pathways				
cbu00230	Purine metabolism	5.20E-18*	2.00E-01	5.40E-06*	1.10E-12*
cbu00010	Glycolysis/Gluconeogenesis	1.00E-13*	3.80E-03*	1.50E-07*	1.50E-07*
cbu00190	Oxidative phosphorylation	3.10E-10*	2.40E-01	1.90E-02*	1.30E-01
cbu00240	Pyrimidine metabolism	5.20E-06*	9.80E-03*	1.80E-08*	5.60E-05*
cbu00564	Glycerophospholipid metabolism	1.80E-05*	6.30E-02	6.00E-02	6.80E-02
cbu00020	Citrate cycle (TCA cycle)	9.20E-04*	-	3.40E-02*	5.30E-02
cbu00071	Fatty acid metabolism	3.10E-03*	-	-	-
cbu00061	Fatty acid biosynthesis	3.20E-03*	1.40E-02*	7.40E-02	7.90E-02
	Biosynthesis of secondary metabolites				
cbu00900	Terpenoid backbone biosynthesis	2.80E-05*	-	-	-
cbu00780	Biotin metabolism	1.00E-04*	1.00E-03*	2.30E-03*	2.40E-06*
cbu00730	Thiamine metabolism	6.10E-04*	7.90E-03*	9.80E-03*	1.30E-05*
	Biosynthesis of amino acids				
cbu00400	Aromatic amino acids biosynthesis	8.40E-08*	-	-	-
cbu00250	Alanine, aspartate and glutamate metabolism	3.40E-05*	-	2.30E-02*	1.20E-03*
cbu00260	Glycine, serine and threonine metabolism	2.20E-04*	1.60E-04*	2.30E-06*	1.10E-04*
	Virulence				
cbu00540	Lipopolysaccharide biosynthesis	5.40E-10*	2.80E-04*	3.70E-05*	1.90E-04*
cbu03070	Bacterial secretion system	7.40E-08*	6.90E-02	-	1.30E-03*
	Repair mechanisms				
cbu03430	Mismatch repair	7.90E-13*	-	3.60E-06*	1.50E-17*
cbu03410	Base excision repair	1.00E-07*	-	3.40E-02*	2.50E-05*
cbu03420	Nucleotide excision repair	8.00E-07*	-	-	4.50E-02*
	Others				
cbu00550	Peptidoglycan biosynthesis	4.00E-13*	-	-	3.30E-03*
cbu02010	ABC transporters	1.00E-04*	-	4.00E-02*	1.10E-03*
cbu02020	Two-component system	3.50E-03*	-	-	-

The *P* value (adjusted by Benjamini-Hochberg method) of regulated pathway is shown for each comparison of 602 strain. Significant pathways (*p* < 0.05) are depicted with *. In vivo (m = Mice spleen), cell culture (cc), cell-free adapting phase passage 1 (p1), cell-free adapting phase passage 2 (p2), cell-free culture model (cf)

**Fig. 4:**
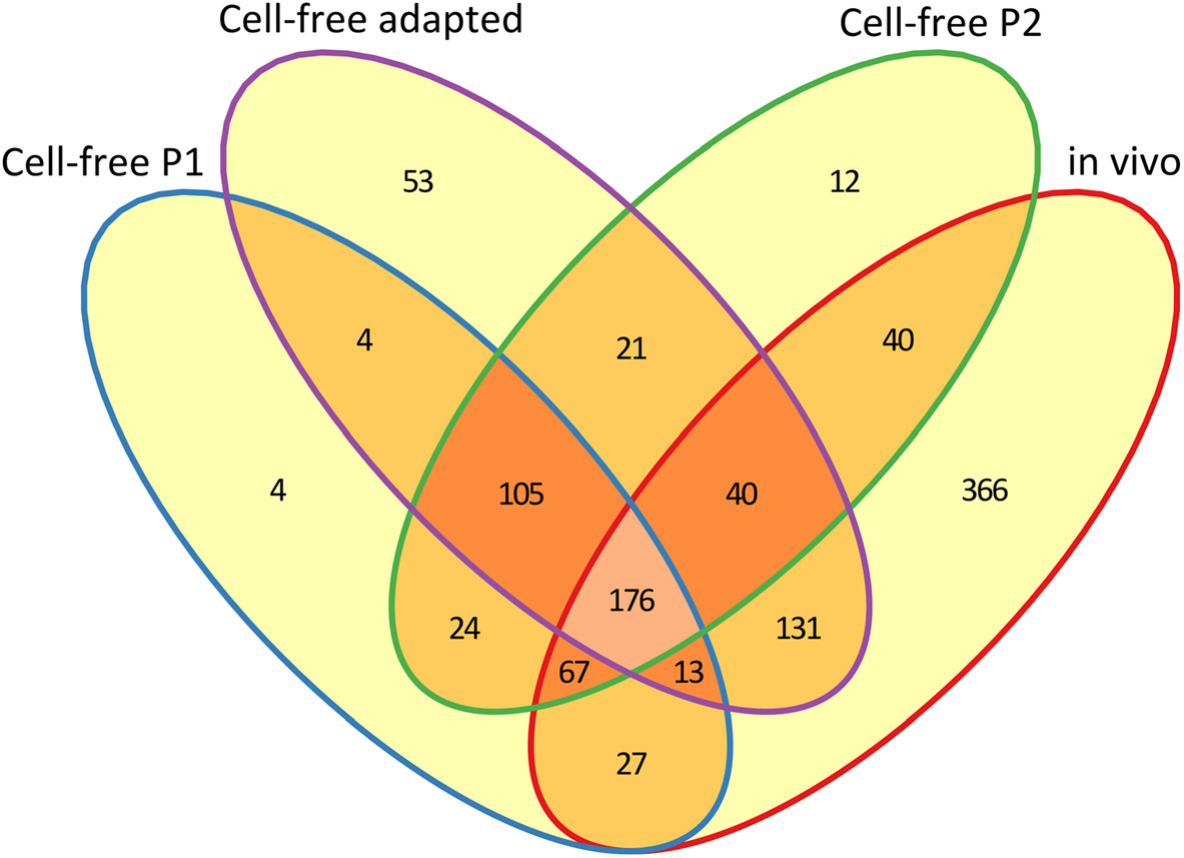
Venn diagram of differentially expressed genes of 602 strain in different culture models. Comparison of differentially regulated genes under in vivo, cell-free adapting serial passages (p1 and p2) and fully adapted culture with respect to in vitro growth in cell culture

To gain insight into the distribution and uniqueness of regulated genes in the different culture models, Venn-diagrams were constructed (Fig. 4). Comparison of differentially regulated genes under in vivo conditions and cell-free culture (including p1, p2 and cell-free adapted) with respect to cell culture showed 176 genes which were similarly regulated between different models and a large number of genes (366 genes) which were specifically regulated in vivo (Fig. 4). These data suggest that gene expression of *C. burnetii* under in vivo conditions is quite different from its in vitro culture models.

Among the in vitro models, comparison between p1 and p2 cultures resulted in differential regulation of 0.6 % of genes only, suggesting that these cultures were highly similar. Comparison of p1 and p2 cultures with cell-free adapted and cell culture showed differential regulation of 15 and 30 % of genes respectively. This indicates that based on the transcriptomic profiles, p1 and p2 cultures are closer to cell-free adapted culture than those from cell culture. The 15 % of genes that were differentially regulated in p1 and p2 compared to cell-free culture were further categorised into functional groups. About 40 % of these differentially regulated genes in p1 and 30 % in p2 were involved in metabolism categories (CEFGHIPQ), showing almost a 10 % decrease in metabolism related genes in p2 within 1 passage, itself in a cell-free environment. This shows that p1 and p2 cultures regulate the genes in a way similar to adapted cell-free culture, most likely due to available resources in the culture media. Thus, the rapid adaptation capabilities of *C. burnetii* were seen when moved from one environment to another.

### *C. burnetii* requires metabolic adaptations for different culture models

**Fig. 5:**
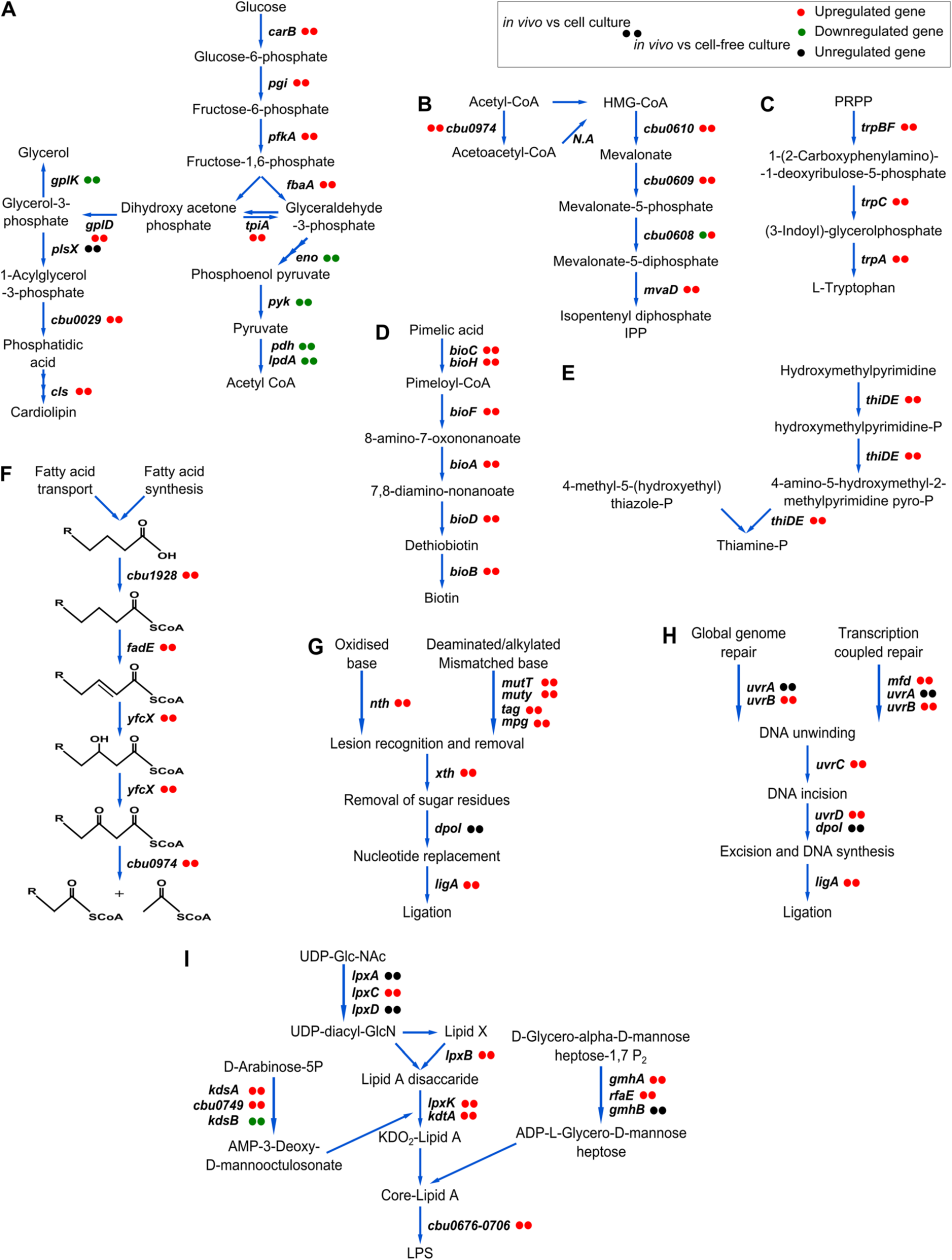
Some metabolic pathways and pathways implicated in virulence and repair mechanisms induced in vivo compared to in vitro cultivation. The pathways were drawn according to KEGG database. Circles next to each gene display its regulation for each condition. **a**–**i** represent significantly up-regulated KEGG pathways under in vivo conditions: **a** Cardiolipin synthesis, **b** Mevalonate pathway, **c** Tryptophan synthesis, **d** Biotin synthesis, **e** Thiamine synthesis, **f** Fatty acid degradation, **g** Base excision repair, **h** Nucleotide excision repair, and **i** LPS synthesis. Reactivity of in vivo vs cell and cell-free cultures are always the same (except cbu0608, due to induction of mevalonate pathway in cell culture similar to in vivo, relative to cell-free culture system)

Striking differences were found between in vivo and in vitro culture models in metabolic pathway regulation. These differences could be attributed to the type of nutrient the bacteria were able to use for metabolism. Under in vivo conditions, genes implicated in glucose transportation (CBU0265), phosphorylation and glycolytic intermediates generation were up-regulated compared to in vitro models [29]. Further, the terminal enzymes of the glycolytic pathway were repressed and genes implicated in phospholipid synthesis were induced, indicating the use of glycolytic intermediates for phospholipid synthesis (Fig. 5a). Among the different phospholipids [30], gene encoding cardiolipins (*cls*), an anionic phospholipid were highly induced in vivo compared to in vitro conditions, indicating an increased synthesis of cardiolipins allowing the bacterium to survive in the highly acidic environment of spleen cell PV [31]. A low level expression of *cls* in cell and cell-free cultures might be sufficient for the bacterium to sustain under in vitro environments, while up-regulation in vivo is possibly required to cope with specific in vivo conditions associated with the acidic environment. In a previous study, cardiolipids were not identified by biochemical analysis of phospholipids in *C. burnetii* grown from in vitro embryonated eggs [32], implying its greater importance under in vivo conditions. These data confirm previous research indicating that the phospholipid content of microorganisms can differ based on the bacterial environment in order to cope with new environmental conditions [33].

Genes implicated in the synthesis of lipids were enhanced in vivo more than under in vitro conditions. Enhanced expression of a putative long-chain fatty acids translocation gene (CBU1242) across the outer membrane of the bacterium was seen, indicating its abundant presence in the PV of host spleen cells. Interestingly, genes coding *de novo* synthesis of fatty acid were also induced, probably to synthesize more fatty acids to meet the overall requirements of the bacterium. The data shows *C. burnetii* can acquire lipids through both *de novo* bacterial synthesis as well as by subversion of host cell pools as indicated previously [30]. Further, the mevalonate pathway, which encodes an isoprenoid backbone (Fig. 5b), [24, 34] and other genes (*uppS, ispB*) that use isoprenoids as substrates were up-regulated in vivo and might help *C. burnetii* in cell wall synthesis and normal growth in host cells [35, 36]. Apart from synthesis of its own lipids, it has been speculated that *C. burnetii* can use host cell cholesterol precursors and convert these into cholesterol. The genome of *C. burnetii* encodes putative Δ24 sterol reductase (CBU1206) the biological role of which is not known, but in general are involved in final stages of cholesterol synthesis in eukaryotic cells. Maintaining such a unique gene in its genome and its up-regulation suggests a critical function for this gene in the bacterium in vivo [37–39]. The role of cholesterol in bacterial pathogenesis is unclear [40, 41], and cell culture of *C. burnetii* lacking cholesterol showed either no effects or negative effects on replication and PV morphology [38, 39]. Sterol reductase could be involved in cholesterol synthesis in limiting sterol conditions. However, since mammalian cells have a high sterol content, the bacteria would not need to synthesize more cholesterol. It is more likely that up-regulation of sterol reductase is involved in generating novel sterol species that have signalling, structural roles or associated with oxidative stress responses [30, 40–43]. Within in vitro models, induction of these genes was seen in cell culture at lower levels than observed in the in vivo system, indicating that the synthesis of these lipids is only intracellular.

The size of the genome of *C. burnetii* is highly reduced (around 2 Mb) comparable to size in other obligate intracellular pathogens. This renders *C. burnetii* auxotrophic for several amino acids or causes it to lack key enzymes in several biosynthetic pathways [24]. Yet under in vivo conditions, several amino acid and oligopeptide transporters, pathways implicated in synthesis of aromatic amino acids (Fig. 5c) [24, 44, 45] and degradation of amino acids (L-serine, L-aspartate, L-glutamine, L-cysteine, L-methionine, L-proline) were highly induced. Taken together, these observations suggest that *C. burnetii* can acquire several amino acids from hosts and synthesizes a few that may be in limited supply due to severe nutritional stress in host cells. A lower level of nutritional stress can be inferred in in vitro conditions, since compared to in vivo only a few transporters are up-regulated in cell culture (CBU1798, CBU0539) and cell-free culture (CBU1130, *artM*).

A large number of genes coding several co-factors (biotin, thiamine, folic acid and Coenzyme A) was also up-regulated in vivo relative to in vitro culture models (Fig. 5d, e). The high level expression of these genes implicated in co-factor synthesis, might indicate lack of readily available co-factors in the spleens of mice. Contrary, it could be possible that the *C. burnetii* genome might not encode co-factor transporters and therefore synthesizes them since they are required for growth under in vivo conditions. The cell and cell-free culture media are rich in co-factors and down-regulation of pathways involved in co-factor synthesis; imply transportation of co-factors by the bacterium. As of now, specific co-factor transporters for these organic co-factors are not reported, but *C. burnetii* encodes several generic transporters in its genome which could potentially transport these co-factors that are crucial for several enzyme reactions involved in amino acid, glucose, lipid metabolism or the regulation of genes.

As the fatty acid degradation pathway was up-regulated in vivo, catabolism of fatty acids appeared to play an important role in energy metabolism. Among the genes implicated in this pathway, the *yfcX* gene was most strongly induced (Fig. 5f). This results in anaerobic degradation of long/ medium chain fatty acids and produces NADH as a major cofactor for energy generation [46]. Genes involved in carbohydrate metabolism (dehydrogenases such as *pdhA, pdhC, lpdA, sucB, sdhA, sdhC*) were repressed under in vivo conditions, indicating an abundance of fatty acids (and fewer carbohydrates) in host cells. In cell and cell-free cultures these carbohydrate metabolism genes were enhanced, suggesting oxidation of glycolytic and TCA intermediates for energy production. The Cytochrome bd oxidase gene (*cydA*) was highly induced under in vivo conditions but at lower levels in cell culture, indicating an increased affinity for oxygen under intracellular conditions [47]. These differences suggest that the kind of nutrients found in the environment affects pathogen manipulation.

In conclusion, under in vivo conditions, the transportation of organic nutrients was up-regulated, showing increased dependence of the bacterium on host-derived products. In vivo, the bacteria also shows enhanced biosynthetic capacities, which are directed to fulfill metabolic requirements that are unavailable directly from the host. In contrast, in vivo the bacterium represses a majority of genes coding for nucleotide synthesis and ribonucleotide reductases*,* which could be connected to the slow replication rate observed compared to in vitro culture conditions. Within in vitro cultures, gene regulation in cell culture were similar to in vivo but at much lower mRNA levels, indicating similar intracellular metabolic adaptations. Hence anaerobiosis, nutrient limitations, inaccessibility of co-factors were prominent environmental conditions encountered by *C. burnetii* during growth in vivo in the mice spleens.

### Enhanced regulation of transporter systems in hosts

Bacterial transporters import essential nutrients, but are also used to import physiological substrates and export toxic molecules, both critical for cell viability, pathogenicity and virulence [48]. Under in vivo conditions, many transporters encoded by *C. burnetii* due to its intracellular life style were probably induced to maintain homeostasis [24]. Among these transporters are, sodium ion/proton exchangers (*shaE, shaA, nhap.1*), which might play a vital role in bacterial cytoplasmic pH homeostasis within the acidic PV. Several predicted drug-efflux systems (CBU0804, CBU0048, CBU0833, CBU1808, CBU1809) were enhanced, providing resistance and removal of toxins and host defensins. Among the metal transporters, gene implicated in inorganic phosphate transport were strongly up-regulated (CBU0014) under in vivo conditions along with up-regulation of phosphate starvation-responsive loci *phoBR,* which is one of the few two-component regulatory system present in *C. burnetii*. Under phosphate starvation, enhanced expression of ATP producing genes was observed, possibly due to increased ATP demand caused by a limited amount of available phosphate [49].

### High degree of oxidative stress in host cells

The generation of oxidative stress agents, which damage key bacterial components, is an innate defense response of host phagocytic cells. Strategies used by *C. burnetii* to avoid reactive substances mainly involve the enzymatic destruction of radicals and DNA repair mechanisms. Among ROS scavenging systems, peroxiredoxin (*ahpD*) and its response regulator (*oxyR*) genes were enhanced under in vivo conditions. Other ROS scavenging enzymes such as functional catalase (*KatE*) are present in only few *C. burnetii* strains. Presence or absence of a functional catalase gene in 602 was assessed by mapping its genome sequences against a strain containing a functional catalase gene (Cbuk_Q154, Accession number: NC_011528.1) as shown previously [15]. The genome sequences of 602 showed the presence of a highly truncated catalase gene as in NM [50]. The absence of a functional catalase gene in 602 and NM might be compensated by peroxiredoxins, which were enhanced under in vivo hosts. Under in vitro conditions, other ROS scavenging enzymes were enhanced in cell culture (*ahpC1, ahpC2*) and cell-free culture (*sodC*), thus indicating an increased peroxidase presence in intracellular hosts and superoxide radicals in the extracellular cell-free system. An induced protein synthesis was observed in the cell-free system, possibly leading to enhanced expression of the glutathione pathway, which restores protein function by reducing oxidized residues. Genes implicated in DNA repair (base excision repair, methyl mismatch repair and nucleotide excision repair) were strongly enhanced under in vivo conditions and collectively repair various DNA damages caused by oxidative stress (Fig. 5g, h) [51–55]. Also the upregulation of *addAB* genes was seen; this is a novel adaptation of *C. burnetii* in its intracellular niche, which mediates DNA repair via homologous recombination [56]. Thus enhanced expression of DNA repair was seen in vivo and at relatively lower rate in cell culture, indicating bacterial response to increased oxidative stress in intracellular environments.

### Induction of virulence genes in vivo

Among the known virulence factors, lipopolysaccharides (LPS) are considered to be the major determinant of the virulent phenotypic expression and infection of *C. burnetii.* Most likely the LPS shields the bacterial cell surface from innate immune recognition [57, 58] and/or the LPS phase-II works as an immunological decoy. Genes encoding structural features of LPS such as lipid A, the core region and the cluster of genes involved in O-antigen synthesis were enhanced under in vivo conditions compared to in vitro systems (Fig. 5i) [59]. ABC transporters implicated in lipid A (MsbA) and O-antigen (CBU0703) transport were strongly induced, indicating the export of LPS components to the outer membrane, where it plays an important role in bacterial virulence [59, 60]. Within the in vitro models, the p1 and p2 cell-free cultures showed similar level of regulation of O-antigen genes as in cell-culture, whereas the adapted cell-free culture (passage *n* > 10) showed a significant down-regulation of these genes, due to their deletion resulting in an on-going transformation into phase II forms [15]. Occurrence of phase II forms through the functional deletion of O-antigen coding genes might be an advantageous feature in the bacteria, allowing it to conserve the metabolic energy required to synthesize complex polysaccharides [61] and instead use energy for other important processes, such as growth or protein synthesis. This would be consistent with the enhanced expression of LPS genes seen in *C. burnetii* under in vivo conditions, and may be important during infection and increase in adaptation capabilities of the bacterium in hosts. Moreover, modifications in transcriptome of *C. burnetii* were seen only in the O-antigen region of high passaged cells, which might possibly result in reduced virulence potential.

Among the other virulence factors “Type IV secretion systems” (T4SS) have been identified as one of the important transfer systems in *C. burnetii* that delivers bacterial effector proteins into the host cytosol and are intimately involved in the pathogenesis of the bacterium. Genome sequencing has revealed the presence of an intact T4SS homologous to *L. pneumophila* Dot/Icm system. To date, only this system has been shown as a virulent determinant essential for the creation of vacuole, establishing a niche for intracellular survival and replication of the bacterium in eukaryotic host cells [24, 62–66]. Previous transcriptional studies on Dot/Icm system in *C. burnetii* has shown high expression of these genes during early stage infections [67, 68]. Under in vivo conditions, the expression of Dot/Icm genes along with their transcription regulator (*pmrA*) were repressed with respect to in vitro models, probably due to later stages of growth of the bacterium in vivo where the infection has already taken place and the function of these genes had already been exerted, resulting in the establishment of replicative vacuole and a productive infection. Although, the transcription of these genes is repressed in later infection stages, decreased protein levels are maintained constantly [68]. Hence secretion of effector proteins of the T4SS required for maintaining an intracellular niche is still possible in the host cells. Under in vivo conditions, T4SS effector proteins such as ankyrin repeat domain containing proteins (*ankC, ankG*) were up-regulated, and have been shown to interfere with the host cell apoptosis pathway [69]. Other effector proteins encoded by plasmid genes (CBU0014, CBU0015, CBU0006) were also up-regulated. These proteins are delivered by T4SS into host cytosol and might play a role in subversion of host cell functions [70]. Under in vitro conditions, the T4SS was up-regulated. In cell culture, this system must be enhanced as the bacteria emerging from PV can re-infect new cells in the culture. Interestingly, this system was also seen to be enhanced in cell-free culture, although the bacterium does not require it as the medium is devoid of cells. Hence, the cell-free in vitro environment might induce the full repertoire of transcriptional responses required for successful pathogenesis of the bacterium for future challenges and transmission.

Others predicted virulence factors implicated in the manipulation of host-cell components and processes were also relatively enhanced under in vivo conditions. Among these were putative genes encoding phospholipase A (CBU0489) which can act on host phospholipids and generate lipid signaling [50, 71], acid phosphatase (CBU0335) which can phosphorylate host proteins and decreases oxidative burst after phagocytosis of the bacterium [67], proteins with eukaryotic like domains (CBUA0014, CBU2078) which can modulate host ubiquitination pathways and disrupting host cell processes during infection [72, 73] and other eukaryotic like protein kinases similar to STPKs (CBU0175, CBU1379) which can directly interfere in host cell signal transduction [50, 74]. Hence, strong regulation of genes involved in manipulating and functionally mimicking the activity of host cell proteins was seen as a virulence property of the bacterium in hosts.

### Stringent responses of *C. burnetii* in hosts

Under environmental stress (such as the intracellular in vivo conditions), bacterial species show stringent responses to escape unfavorable conditions. Under in vivo conditions, genes implicated in alarmone degradation were regulated (down-regulation of *spoT* and up-regulation of *relA*) which results in accumulation of unusual guanosine nucleotides (p)ppGpp, a hallmark of stringent response in several bacterial species [75]. In several bacterial species stringent responses were shown to play a role in sporulation, virulence and long-term persistence, usually in response to environmental stress [75, 76]. For example, in *L. pneumophila*, a close relative of *C. burnetii,* the stringent pathways are important factors in modulating the virulent attributes that help its survival in the host [77]. The role of these regulatory networks in *C .burnetii* is not known yet and differential regulation of these genes under in vivo conditions at later infection stages (Day 20) may result in a greater benefit to the survival of the bacteria, which would be an interesting area of study in need of further investigation.

Additional microarray experiments performed in parallel with NM reference strain revealed similar gene expression patterns in vivo compared to in vitro models (data not shown) indicating that the trends described by the Dutch 602 outbreak field strain are likely to prove relevant to other *C. burnetii* strains more generally. Therefore, the microarray data increases our knowledge regarding pathogen adaptation to host micro environments, selective pressures in these environments and bacterial factors responsible for evading host responses. Finally, the microarray data can be used as a guide to screen and select genes encoding putative membrane proteins and other proteins that have antigenic properties among the differentially regulated genes during varying conditions. This might provide a framework for new possibilities to investigate and identify virulent factors important in the design of vaccines.

## Conclusions

This is the first report on *C. burnetii* genome-wide transcriptome profiles isolated from in vivo and in vitro culture models. The data provide insight into pathways associated with bacterial adaptation to varying environmental conditions and identify potential virulent factors based on the mRNA levels of genes measured by microarray approach. Transcriptome differences in *C. burnetii* were profound between in vivo and in vitro culture models. Within the in vitro culture systems, the expression profile of *C. burnetii* from cell culture were closely related to expression profiles from the in vivo model (Fig. 2). This might be due to its intracellular nature and low nutrient availability in cell culture compared to free availability of nutrients in the more artificial cell-free culture model. The cell-free culture adapting passages (p1 and p2) expression profiles migrated towards cell-free adapted strains indicating the ability of the bacterium to adapt promptly to available resources in the environment.

Between the different models, major differential gene regulations was primarily observed in metabolic processes of the bacteria. Under in vivo conditions, the exciting observations in our study were; enhanced expression of anionic phospholipid coding gene (*cls*) for maintenance of acidic pH at outer membrane surface; organic molecule transportation indicating non-starvation of the bacteria; phosphate limitation as an important intracellular signal, and enhanced DNA repair mechanisms to combat against oxidative stress in host cells. We have also been able to provide evidence that based on the observed gene-regulation, lipids play a very important role in several biosynthetic processes of *C. burnetii* in vivo and are the primary sources for anaerobic energy metabolism, possibly due to their abundant presence in mice spleens. However, carbohydrate metabolism was observed under in vitro culture systems indicating type and availability of nutrients were important determinants for resulting expression patterns of *C. burnetii* in different models. Relative to in vitro conditions, the in vivo environment significantly enhanced expression of virulence genes such as LPS-linked genes and factors implicated in host-cell modulations (inhibition of apoptosis, evading defense mechanisms etc.). This capability to modulate host response to infection seems to be a key factor for *C. burnetii* survival in vivo.

## Methods

### Bacterial strains and culture models

*C. burnetii* strain 602 was isolated from aborted placenta of goats during a Q fever outbreak and initially propagated on Buffalo Green Monkey (BGM) cells (European Collection of Cell Cultures, Salisbury) as described previously [15, 20]. The strain was genotyped by MLVA as CbNL01, a predominant genotype during the outbreak. The 602 and Nine Mile RSA493 (NM) reference strain were propagated in different models explained below.

### Mice spleen model

The spleens from mice infected with *C. burnetii* 602 and NM strains were used as in vivo model to study bacterial gene expressions. Animal experiments were conducted using 7 week old Specific-Pathogen-Free Swiss female OF1 mice (Charles River, l’Arbresle). The mice were housed under sterile conditions in biosecurity level 3 facilities. After a week of acclimatization, infections were performed with 3 Swiss OF1 mice per strain. The mice were inoculated intraperitoneally with 0.2 ml PBS suspension of *C. burnetii* (grown in BGM cell culture) with each mouse receiving a dose of 10^7^ genome copy equivalents as determined by quantitative PCR [20]. Twenty days after inoculation the mice were euthanized, spleens were harvested aseptically and immediately frozen in liquid nitrogen and then at −80 °C for RNA isolation. Animal experiments were approved by the animal experiment commission of the Central Veterinary Institute part of Wageningen UR, and conducted in accordance with the Dutch regulations on animal experimentation.

### In vitro culture models

The *C. burnetii* strains used in the study were propagated in cell and cell-free cultures in triplicate from the low passage stock cultures stored at −80 °C.

### Cell culture model

For the cell culture model, BGM cells with EMEM culture media without antibiotics (10 % bovine serum albumin, 1 % NEAA, 1 % glutamax) were used to propagate *C. burnetii* from stock cultures (passage *n* = 10–12 in BGM cell culture) until stationary growth phase [20].

### Cell-free culture model

For the cell-free culture, the protocol previously described [23] was used to propagate *C. burnetii* (passage *n* = 10–12 in cell-free culture) until stationary growth phase. Two serial passages (p1 and p2) of the strains from cell to cell-free culture model were also performed in addition and were included in the study.

### *C. burnetii* specific RNA isolation

The frozen spleens were thawed on ice and a piece of the tissue was homogenized (PowerGen 125, Fischer Scientific, Pittsburgh) in trizol reagent (Ambion, Austin) with a disposable plastic micro pestle (OMNI-Tip plastic homogenizing probes, OMNI international, Kennesaw) and used for RNA isolation. The in vitro cell culture supernatants were centrifuged at 1200 rpm, 4 °C for 10 min to remove cell debris. These processed supernatants of cell and the cell-free cultures were centrifuged at 16,000 rpm, 4 °C for 30 min to pellet the bacterial cells and subsequently treated with trizol for RNA isolation. Total RNA was isolated by using Direct-zol RNA MiniPrep Kit (Zymo Research, Irvine) as per manufacturer’s instructions. All samples were DNAse treated prior to RNA isolation. The host RNA from total spleen RNA were selectively removed by MICROBEnrich kit (Ambion, Austin) followed by RNA clean and Concentrator-5 kit (Zymo Research, Irvine) for RNA clean-up and concentration.

### *C. burnetii* specific microarray

Custom gene expression microarrays, 8×15 K, designed by Agilent (Agilent Technologies, Santa Clara), consisted of eight arrays per slide with duplicates of 7500 probes. All the probes were user-designed by eArray web-based probe design tool (https://earray.chem.agilent.com/earray) based on the complete gene repertoire of *C. burnetii* (NCBI, Accession numbers: AE016828.2, CP000733.1, CP001020.1, CP001019.1, CP001021.1 and draft genomes of Dutch *C. burnetii* strains). 1 to 4 probes of 120 bp in length were designed per gene such that the probes evenly distributed across the gene.

### RNA amplification, labelling and hybridization

Poly A tails were tagged to purified bacterial total RNA (500 ng) from all the culture models with a Poly A Polymerase Tailing kit (Epicenter Illumina company, Madison) as per manufacture instructions. Poly A tailed RNA (250 ng) were prepared for amplification and labelling using the Low Input Quick Amp Labeling Kit from Agilent following the detailed kit protocol. Cyanine 3–labelled cRNA was purified using RNeasy mini kit (Qiagen, Hilden). Labelled cRNA was examined with the Nanodrop ND-1000 (Thermo Fisher Scientific, Wilmington) to assess its concentration and quality. Hybridization and post-hybridization washes were conducted according to Agilent’s one-color microarray-based gene expression analysis protocol (Version G4140_90042).

### Data and statistical analysis

Microarray slides were scanned using a Surescan High Resolution DNA microarray scanner (Agilent Technologies, Santa Clara). Data was obtained through Agilent’s feature extraction software (Version 10.7.3.1) and loaded in Genespring GX (Version 12.6.1) where signal intensity was log_2_-transformed and median normalized for all triplicate samples before analysis. PCA plots (based on correlation coefficients obtained from pair-wise comparisons of samples) and clustering of microarray data (based on Euclidian distance and Ward’s linkage parameters) were analysed using Genespring GX to display relative differences across samples. Differentially regulated genes between different culture models were determined by 2-way ANOVA (*p* < 0.05, adjusted by the Benjamini and Hochberg false discovery rate algorithm) and a cut-off of 2 for the fold change was identified as significantly differential expression. These differentially regulated genes from different comparisons were plotted by means of a Venn diagram using Vennerable package (Version 2.0). David (http://david.abcc.ncifcrf.gov/) and KeggArray software (http://www.genome.jp/kegg/) was used for pathway analysis and to obtain *P* values of significantly regulated pathways for each comparison of 602 and NM strains against *Coxiella burnetii* RSA 493 (organism code: cbu) KEGG pathway database.

## Additional files

Additional file 1: Figure S1. Changes in splenomegaly and bacterial genome equivalents present in the spleens of mice infected with 602 and NM *C. burnetii* strains at 7 and 20 days p.i. A) Bacterial genome equivalents of total spleens are evaluated by qPCR quantification and expressed as log2 transformed values, B) Degree of splenomegaly are expressed as the percentage of spleen weight compared with the body weight. The results are indicated as means ± standard deviation, * indicates p-values smaller than 0.05. (TIFF 265 kb) Additional file 2: Table S1. List of genes differentially regulated in vivo and cell-free culture with respect to cell culture in vitro model of 602 strain, after passing statistical significance filter and showing more than two fold change in expression. All the genes are arranged based on COG functions. A positive value indicates that the gene is up-regulated, a negative value indicates that the gene is down-regulated and – indicates no difference compared to cell culture in gene regulation under the respective condition. (XLSX 204 kb)
