# Supplementary figures and images for: Major differential gene regulation in Coxiella burnetii between in vivo and in vitro cultivation models
Source: BMC Genomics. 2015 Nov 16;16:953. doi: 10.1186/s12864-015-2143-7 (PMC4647677; doi:10.1186/s12864-015-2143-7)

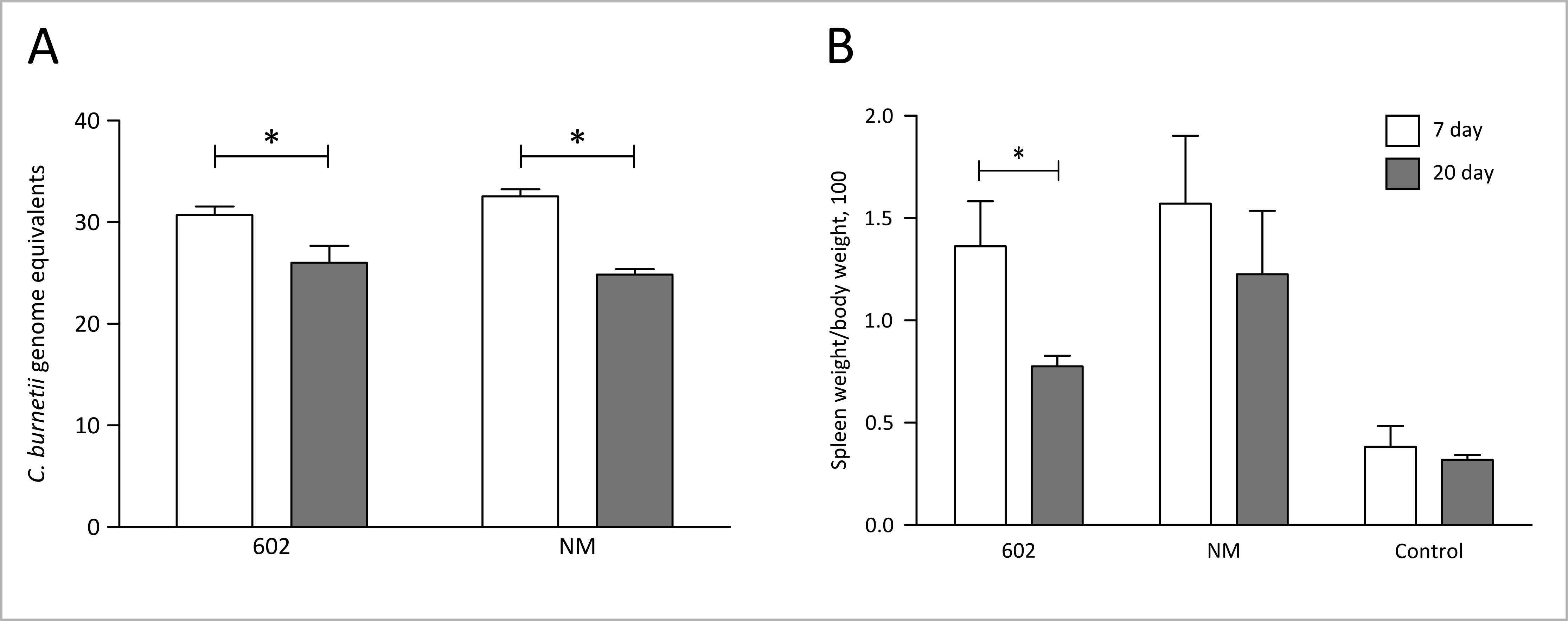

Supplement: Additional file 1: Figure S1. — Changes in splenomegaly and bacterial genome equivalents present in the spleens of mice infected with 602 and NM C. burnetii strains at 7 and 20 days p.i. A) Bacterial genome equivalents of total spleens are evaluated by qPCR quantification and expressed as log2 transformed values, B) Degree of splenomegaly are expressed as the percentage of spleen weight compared with the body weight. The results are indicated as means ± standard deviation, * indicates p-values smaller than 0.05. (TIFF 265 kb) [file 12864_2015_2143_MOESM1_ESM.tif]
